# Systemic functional enrichment and ceRNA network identification following peripheral nerve injury

**DOI:** 10.1186/s13041-018-0421-4

**Published:** 2018-12-17

**Authors:** Tianmei Qian, Chunlin Fan, Qianyan Liu, Sheng Yi

**Affiliations:** 1 Key Laboratory of Neuroregeneration of Jiangsu and Ministry of Education, Co-innovation Center of Neuroregeneration, Nantong University, 19 Qixiu Road, Nantong, Jiangsu Province People’s Republic of China 226001; 2 Department of Respiratory and Critical Care Medicine, Affiliated Hospital of Nantong University, Nantong, Jiangsu China

**Keywords:** Peripheral nerve injury, RNA sequencing, lncRNA, CeRNA, Bioinformatic analysis

## Abstract

Peripheral nerve injury is a worldwide clinical issue that impacts patients’ quality of life and causes huge society and economic burden. Injured peripheral nerves are able to regenerate by themselves. However, for severe peripheral nerve injury, the regenerative abilities are very limited and the regenerative effects are very poor. A better understanding of the mechanisms following peripheral nerve injury will benefit its clinical treatment. In this study, we systematically explored the dynamic changes of mRNAs and long non-coding RNAs (lncRNAs) in the injured sciatic nerve segments after nerve crush, identified significantly involved Gene ontology (GO) terms and Kyoto Enrichment of Genes and Genomes (KEGG) pathways, and innovatively analyzed the correlation of differentially expressed mRNAs and lncRNAs. After the clustering of co-expressed mRNAs and lncRNAs, we performed functional analysis, selected GO term “negative regulation of cell proliferation”, and constructed a competing endogenous RNA (ceRNA) network of LIF and HMOX1 gene in this GO term. This study is the first to provide a systematic dissection of mRNA-microRNA (miRNA)-lncRNA ceRNA network following peripheral nerve injury and thus lays a foundation for further investigations of the regulating mechanisms of non-coding RNAs in peripheral nerve repair and regeneration.

**Electronic supplementary material:**

The online version of this article (10.1186/s13041-018-0421-4) contains supplementary material, which is available to authorized users.

## Introduction

Peripheral nerve injury, resulting from a variety of different reasons such as mechanical compression, ischemia, penetrating injury, stretch injury, and cold injury, may seriously affect patients’ quality of life and cause huge society and economic burden [1]. It is reported that peripheral nerve injury affects about 1.5–2.8% of trauma patients and often leads to life-long morbidity and disability [2, 3]. In the United States, about 360,000 patients are suffering from upper extremity paralytic syndromes annually and more than $150 billion is used to treat peripheral nerve injury every year [4, 5].

Different from the central nervous system which has a very limited ability to regrowth, the peripheral nervous system obtains an intrinsic regenerative power [6]. In spite of this, the regenerative outcomes of injured peripheral nerves, especially peripheral nerves with severe defects and long nerve gaps, are generally poor and incomplete. Surgical nerve repair, including direct suturing and the transplantation of autologous nerve graft or tissue engineered nerve graft, improves the functional recovery of injured peripheral nerves [7]. However, the repairing effects of these therapeutical strategies are far from satisfactory. Gaining a better understanding of the cellular and molecular mechanisms underlying peripheral nerve injury and regeneration may contribute to the clinical treatment of peripheral nerve injury.

High-throughput screenings, such as microarrays and sequencing, are advanced large scale technologies for genome-wide analysis. Sequencing directly detects transcripts and has many advantages such as low background noise, large dynamic range, and high reproducibility [8, 9]. Besides the systematic identification of expressed profiles of known mRNAs, the application of sequencing also benefits the identifications of unannotated transcripts and non-coding RNAs, such as microRNAs (miRNAs), long non-coding RNAs (lncRNAs), and circular RNAs (circRNAs) [10–13]. In a previous study, by using RNA deep sequencing, we obtained the global transcriptome profiles of lesioned rat sciatic nerves at 0, 1, 4, 7, and 14 days after nerve crush. By using Ingenuity pathway analysis, we analyzed differentially expressed mRNAs and revealed key biological functions and canonical signaling pathways [14].

In the current study, with the joint use of Euclidean distance calculation, hierarchical clustering, principal component analysis, Gene ontology (GO), and Kyoto Enrichment of Genes and Genomes (KEGG), we further systematically determined the dynamic genetic changes following peripheral nerve injury. Besides a deeper investigation of differentially expressed mRNAs, we also identified differentially expressed lncRNAs, combined differentially expressed lncRNAs with differentially expressed mRNAs, and constructed correlated competing endogenous RNA (ceRNA) networks of LIF gene and HMOX1 gene based on miRWalk-validated miRNA-mRNA interaction and TargetScan-predicted miRNA-lncRNA interaction.

## Materials and methods

### RNA deep sequencing and data access

RNA sequencing was performed by using Illumina HiSeq™ 2000 and was described in the previous publication [14]. Briefly, rat sciatic nerve segments were collected from a total of 30 adult male Sprague-Dawley (SD) rats weighting 180–220 g at 0, 1, 4, 7, and 14 days after nerve injury. Total RNAs were extracted from sciatic nerve segments and purified. mRNAs and lncRNAs were fragmented into short pieces to synthesize cDNAs. cDNA fragments were purified, connected with adaptors, and used as templates for PCR amplification. Obtained raw reads subjected to quality control to collect clean reads by removing dirty reads with contain adaptors, high unknown bases, or low quality. Sequencing data were uploaded to NCBI database (accession number PRJNA394957; SRP113121).

### Screening of significantly differentially expressed RNAs

The expression levels of both mRNAs and lncRNAs were calculated by using the Reads per kilobase transcriptome per million mapped reads (RPKM) method. The expression levels of mRNAs and lncRNAs at 1, 4, 7, and 14 days after rat sciatic nerve crush injury were compared to their expression levels at 0 day. RNAs with fold change> 10 and false discover rate (FDR) < 0.001 were screened and considered as significantly differentially expressed.

### Bioinformatic analysis

Significantly differentially expressed RNAs at 1, 4, 7, and 14 days after nerve injury were analyzed by using the Venny 2.1.0 software (http://bioinfogp.cnb.csic.es/tools/venny/index.html) [15, 16] to visualize the intersection of differentially expressed RNAs at each time point. Euclidean distance calculation and hierarchical clustering were performed by using the HeatMapImage GenePattern module to illustrate the temporal expression patterns of differentially expressed RNAs. Principal component analysis was performed by using the Population Principal Component Analysis software (Harvard Medical School, MA, USA) to display the similarity of differentially expressed RNAs at different time point. Database for Annotation, Visualization, and Integrated Discovery (DAVID) bioinformatic resource (https://david.ncifcrf.gov/) was used to identify enriched GO terms and KEGG pathways.

Significantly differentially expressed mRNAs and lncRNAs were subjected to the calculation of Pearson correlation coefficient. mRNAs and lncRNAs with Pearson correlation coefficient index> 0.9 and adjusted *p*-value< 0.1 were considered as co-expressed. K-means clustering was conducted and GO and KEGG analysis was performed to enrich GO terms and KEGG pathways with *p*-value< 0.05 in each cluster. The binding relationships of mRNAs and miRNAs were analyzed and validated by using the miRWalk software (http://zmf.umm.uni-heidelberg.de/apps/zmf/mirwalk2/gopub.html). GO terms with more than one validated gene were screened and GO term “negative regulation of cell proliferation” was selected for the construction of ceRNA network. The binding relationships of miRNAs and lncRNAs were predicted by using TargetScan software (http://www.targetscan.org/vert_72/). The interactions of mRNAs LIF, HMOX1, validated miRNAs miR-494-3p, let-7e-5p, let-7a-5p, let-7d-5p, and predicted lncRNAs were analyzed and corresponding ceRNA network was built.

### Animal surgery

To validate outcomes from bioinformatic analysis, we obtained a total of 45 adult male SD rats (180–220 g) from the Experimental Animal Center of Nantong University and performed sciatic nerve crush injury as previously described [14]. Briefly, after anaesthetization, rat sciatic nerve at 10 mm above the bifurcation into the tibial and common fibular nerves was crushed with a forceps at a force of 54 N for 30 s with 10 s for each time. Rats were sacrificed by cervical dislocation at 1, 4, 7, and 14 days after surgery. Rats underwent sham-surgery were used as 0 day control. All the experimental procedures involving animals were conducted in accordance with Institutional Animal Care guidelines of Nantong University and approved ethically by the Administration Committee of Experimental Animals, Jiangsu, China.

### Quantitative RT-PCR

Sciatic nerve segments at the crush site (different samples from those for RNA deep sequencing) were collected to extract total RNAs by using Trizol Reagent (Life Technologies, Carlsbed, CA, USA). Isolated RNAs were reverse transcribed to cDNAs by using Prime-Script Reagent Kit (TaKaRa, Dalian, Liaoning, China) or TaqMan MicroRNA Reverse Transcription Kit (Applied Biosystems, Foster City, CA, USA). cDNAs were amplified with QuantiNova SYBR Green PCR Kit (Qiagen, Valencia, CA, USA) on an Applied Biosystems Stepone real-time PCR System (Applied Biosystems, Foster City, CA, USA). Relative abundances of mRNAs, miRNAs, and lncRNAs were determined by using the ΔΔCt method. GAPDH was used as the reference gene for the quantifications of mRNAs and lncRNAs and U6 was used as the reference gene the quantifications of miRNAs. Primers used were listed in Additional file 1: Table S1.

### Statistical analysis

Statistical analyses were performed by using GraphPad Prism 6.0 (GraphPad Software, Inc., La Jolla, CA, USA). One-way analysis of variance (ANOVA) and Dunnett’s multiple comparisons test were used for comparison between groups. Numerical data were presented as mean ± SEM and a *p*-value< 0.05 was considered as statistically different.

## Results

### Identification of significantly differentially expressed RNAs after sciatic nerve injury

Our previously obtained sequencing data (SRP113121) discovered a total of 38,967 RNAs (including mRNAs and lncRNAs) in rat sciatic nerve segments with 35,728, 38,024, 36,847, 37,513, and 36,403 RNAs at 0, 1, 4, 7, and 14 days, respectively [14]. After the comparison of the expressions of RNAs in injured rats with those at 0 day, a total of 22,498 RNAs were found to be differentially expressed during the time course (fold change> 2 or < − 2, FDR < 0.001). The vast numerical RNAs increased the difficulty of the discovery of critical information. Therefore, we filtered sequencing data, selected RNAs with fold change> 10 or < − 10 and FDR < 0.001 as compared with 0 day control, and designated these RNAs as significantly differentially expressed RNAs. A full list of all significantly differentially expressed mRNAs and lncRNAs were shown in Additional file 2: Table S2.

**Fig. 1:**
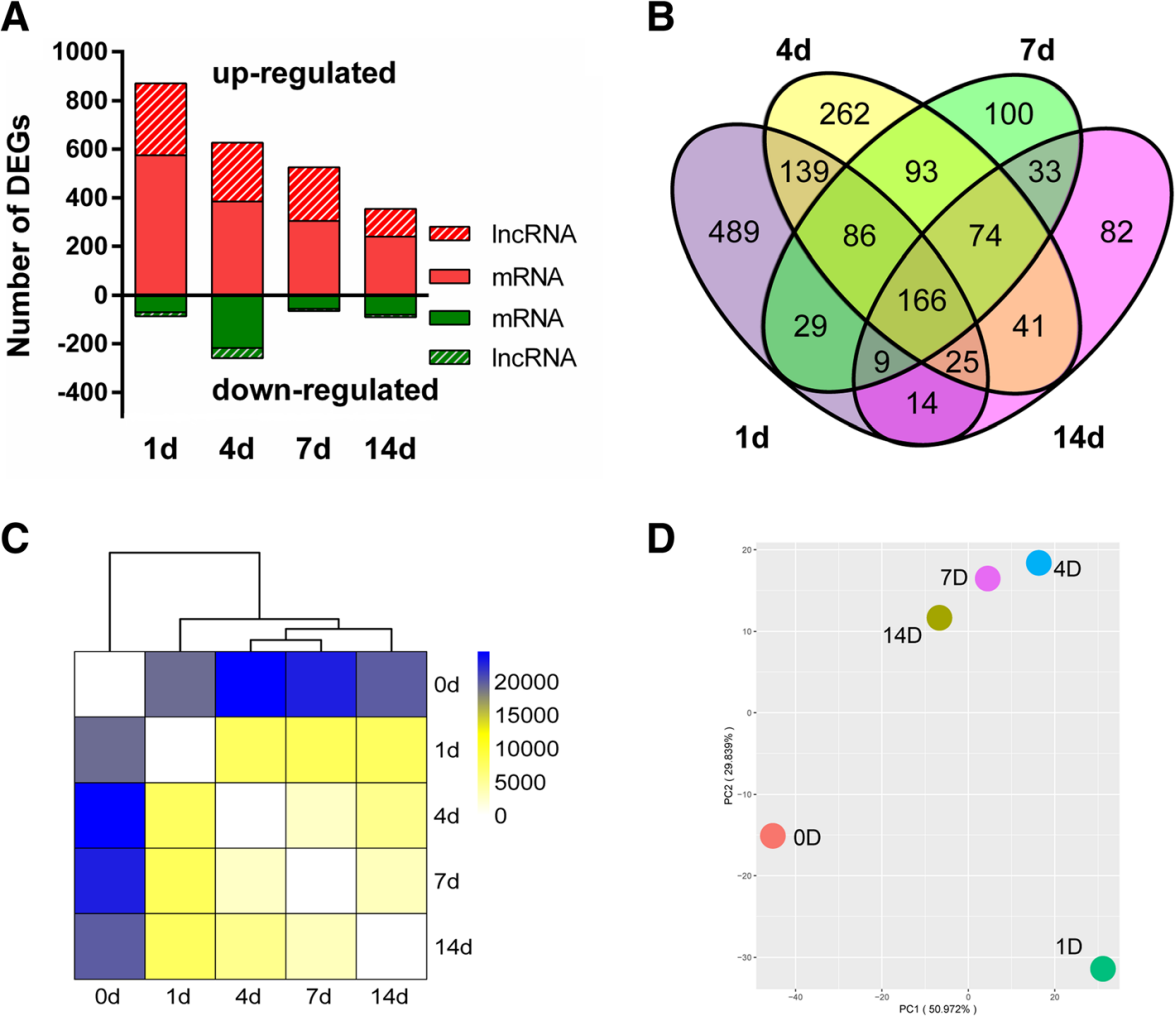
Overview of significantly differentially expressed RNAs in the sciatic nerve segments after injury. **a** The bar graph of significantly differentially expressed mRNAs and lncRNAs at 1, 4, 7, and 14 days after sciatic nerve injury. RNAs with fold change> 10 or < − 10 and FDR < 0.001 as compared with 0 day control were considered as significantly differentially expressed. DEG, differentially expressed genes. Red color indicated up-regulated RNAs while green color indicated down-regulated RNAs. **b** The Venn diagram of significantly differentially expressed mRNAs and lncRNAs at 1, 4, 7, and 14 days after sciatic nerve injury. **c** Euclidean distance and hierarchical clustering of RNAs at 0, 1, 4, 7, and 14 days after sciatic nerve injury. **d** Principal component analysis of RNAs at 0, 1, 4, 7, and 14 days after sciatic nerve injury

It was demonstrated that compared with 0 day control, at 1 day after nerve injury, 575 mRNAs and 295 lncRNAs were up-regulated while 70 mRNAs and 17 lncRNAs were down-regulated. Slightly smaller numbers of RNAs were significantly differentially expressed at 4, 7, and 14 days after nerve injury. At 14 day after nerve injury, only 354 RNAs (240 mRNAs and 114 lncRNAs) were up-regulated and 90 RNAs (80mRNAs and 10 lncRNAs) were down-regulated (Fig. 1a). Significantly differentially expressed RNAs during the time course were further analyzed and illustrated by the Venn diagram. A total of 166 RNAs were commonly up-regulated or down-regulated at all time points while many RNAs were only significantly differentially expressed at one single time point (Fig. 1b). Differentially expressed RNAs were then subjected to cluster analysis to determine the similarity of RNA expression profiles during the time course. Euclidean distance, hierarchical clustering, and principal component analysis demonstrated that the RNA expression patterns in 0 and 1 day were quite different while the RNA expression patterns in 4, 7, and 14 day after nerve injury showed certain consistency (Fig. 1c & d).

### Functional enrichment analysis of differentially expressed mRNAs

**Fig. 2:**
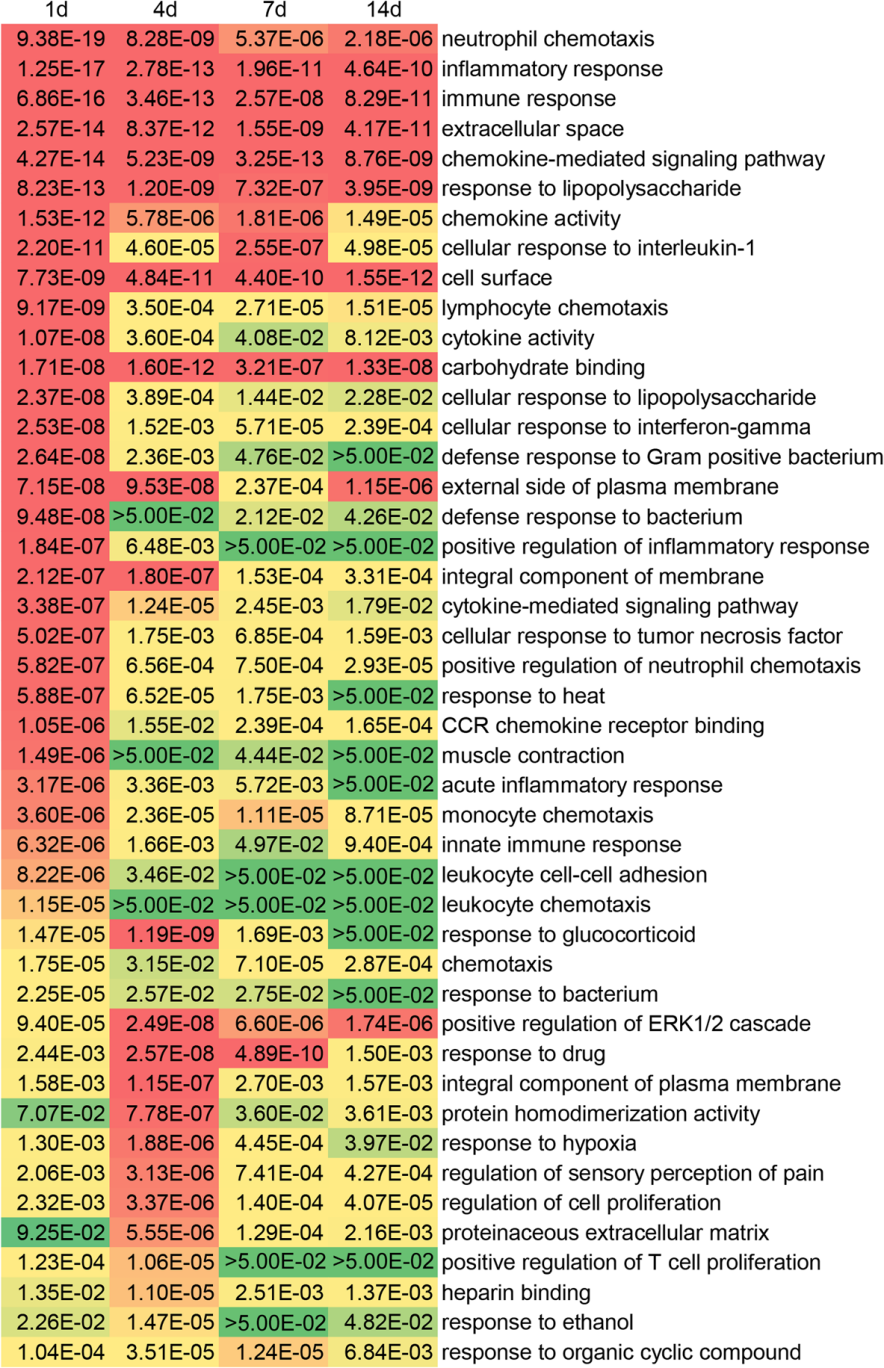
Enriched GO terms of significantly differentially expressed mRNAs in the sciatic nerve segments after injury. GO cellular component, molecular function, and biological process terms with *p*-value< 0.05 and FDR < 0.05 in 1, 4, 7, or 14 days after sciatic nerve injury were screened. The *p*-values of GO terms were listed. GO terms with low *p*-values were labeled in red color while GO terms with high *p*-values were labeled in green color

Significantly differentially expressed mRNAs were then categorized to GO terms and KEGG pathways to discover possible biological functions and pathways involved in peripheral nerve injury. A full list of GO biological process, cellular component, and molecular function terms with a *p*-value< 0.05 at 1, 4, 7, and 14 days after nerve injury was provided in Additional file 3: Table S3. GO terms with a *p*-value< 0.05 and FDR < 0.05 at least one time point were enriched, listed, ranked with *p*-value and FDR from day 1 to day 14, and presented in color gradation (Fig. 2). It was observed that mass GO terms were dramatically involved at 1 day after nerve injury while less GO terms were significantly enriched at later time points. For GO cellular component terms, extracellular space, cell surface, and external side of plasma membrane were the top three enriched terms. For GO molecular function terms, chemokine activity, cytokine activity, and carbohydrate binding were the top three enriched terms. A larger number of GO biological process terms were involved. Notably, many of these enriched GO biological process terms were associated with immune and inflammatory response (neutrophil chemotaxis, inflammatory response, immune response, cellular response to interleukin-1, lymphocyte chemotaxis, cellular response to interferon-gamma, defense response to Gram-positive bacterium, defense response to bacterium, positive regulation of inflammatory response, cellular response to tumor necrosis factor, positive regulation of neutrophil chemotaxis, acute inflammatory response, monocyte chemotaxis, innate immune response, leukocyte cell-cell adhesion, leukocyte chemotaxis, response to bacterium, and positive regulation of T cell proliferation). Many cell signaling pathway-related GO biological process terms, for example, chemokine-mediated signaling pathway, cytokine-mediated signaling pathway, positive regulation of ERK1 and ERK2 cascade, were also enriched.

**Fig. 3:**
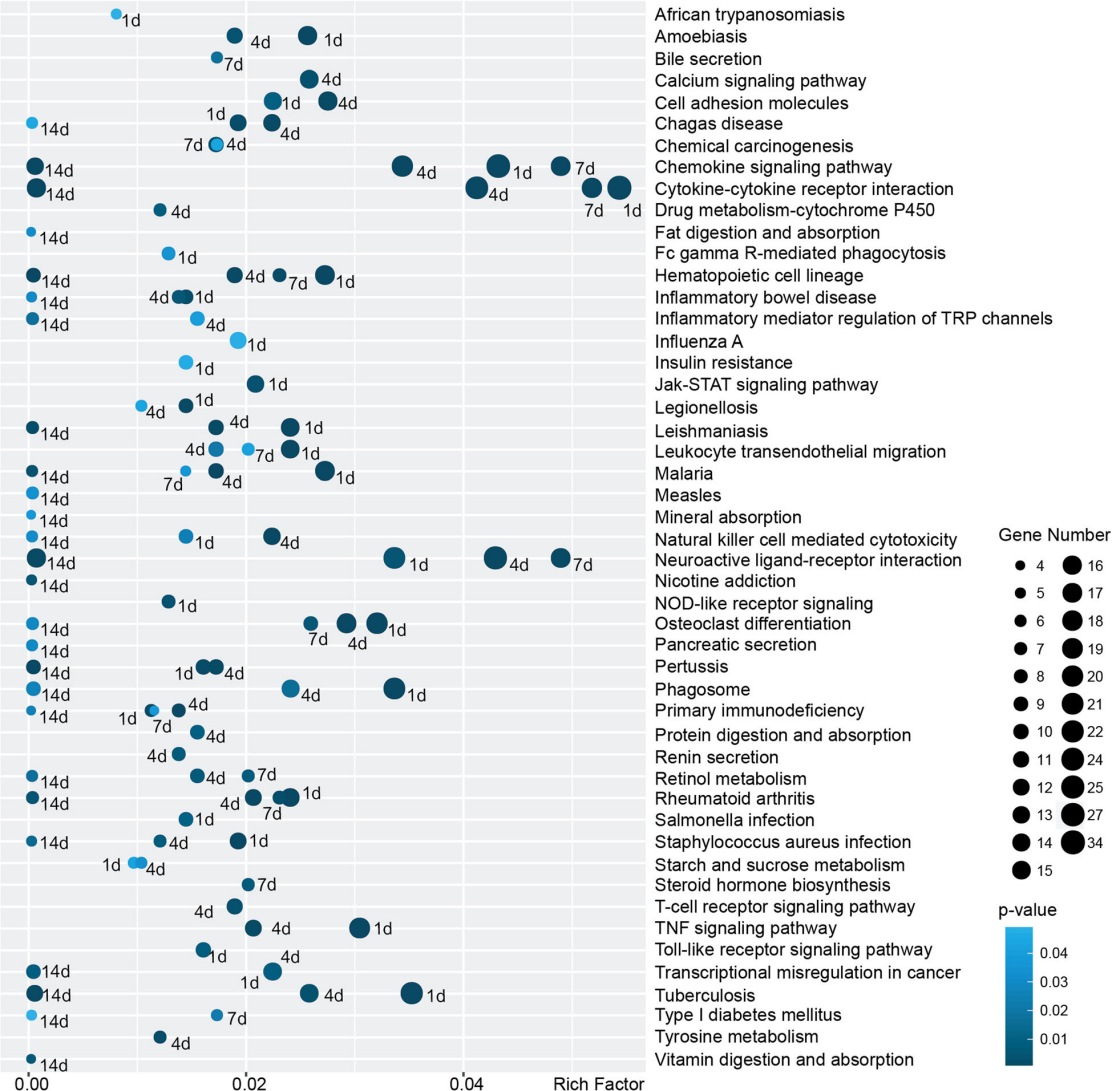
Enriched KEGG pathways of significantly differentially expressed mRNAs in the sciatic nerve segments after injury. KEGG pathways with *p*-value< 0.05 in 1, 4, 7, or 14 days after sciatic nerve injury were screened and listed. The number of significantly differentially expressed genes in each KEGG pathway and relevant Rich Factor were shown

To further investigate the involvement of cell signaling pathways in peripheral nerve injury, KEGG pathway analysis was conducted and critical signaling pathways in peripheral nerve injury were identified (Fig. 3). A total of seven signaling pathways, including chemokine signaling pathway, cytokine-cytokine receptor interaction, hematopoietic cell lineage, malaria, neuroactive ligand-receptor interaction, osteoclast differentiation, and rheumatoid arthritis, were significantly activated at all time points after nerve injury. And signaling pathways chemokine signaling pathway, cytokine-cytokine receptor interaction, and neuroactive ligand-receptor interaction obtained large gene numbers and high Rich Factor, implying their essential roles in peripheral nerve injury.

### Co-expression of significantly differentially expressed mRNAs and lncRNAs

**Fig. 4:**
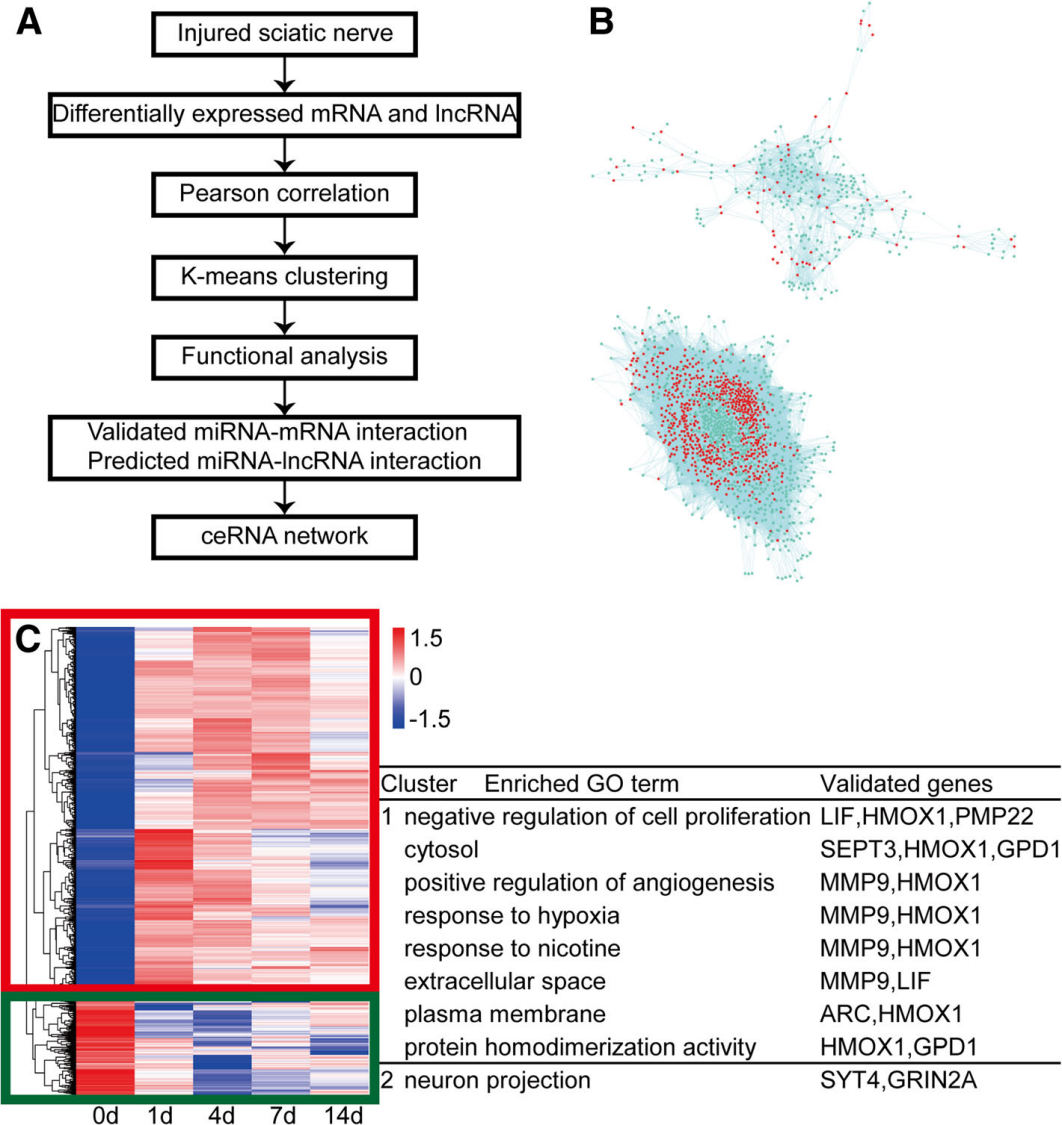
Coexpression analysis of significantly differentially expressed mRNAs and lncRNAs in the sciatic nerve segments after injury. **a** Schematic depiction of coexpression analysis of significantly differentially expressed mRNAs and lncRNAs and the construction of mRNA-miRNA-lncRNA ceRNA network. **b** Person correlation network of significantly differentially expressed mRNAs and lncRNAs. mRNAs were labeled in cyan color while lncRNAs were labeled in red color. **c** K-means clustering of co-expressed mRNAs and lncRNAs. Enriched GO terms and involved validated genes were listed

Besides the functional analysis of mRNAs, we further determined the correlation of significantly differentially expressed mRNAs and lncRNAs, aiming to characterize mRNA-miRNA-lncRNA ceRNA networks in peripheral nerve injury (Fig. 4a). Person correlation analysis showed that many significantly differentially expressed mRNAs and lncRNAs were correlated with each other (Fig. 4b). Co-expressed mRNAs and lncRNAs were then subjected to K-means clustering to group RNAs with different temporal expression patterns. Two clusters of RNAs (boxed by different colors) were categorized and functional enriched to GO terms and KEGG pathways to discover critical biological activities of co-expressed mRNAs and lncRNAs (Fig. 4c). By setting a cutoff of *p*-value< 0.05, we identified a total of 317 activated GO terms and KEGG pathways in cluster 1 and a total of 130 activated GO terms and KEGG pathways in cluster 2 (Additional file 4: Table S4). We further studied involved mRNAs in these GO terms and KEGG pathways and determined the validated binding relationships between mRNAs and miRNAs by using the miRWalk software. A total of 54 GO terms in cluster 1 and 10 GO terms in cluster 2 were found to have mRNAs with validated miRNA binding relationships (Additional file 5: Table S5). Enriched GO terms with more than one validated mRNA were screened and listed (Fig. 4c).

### Construction and examination of LIF and HMOX1-associated ceRNA network

**Fig. 5:**
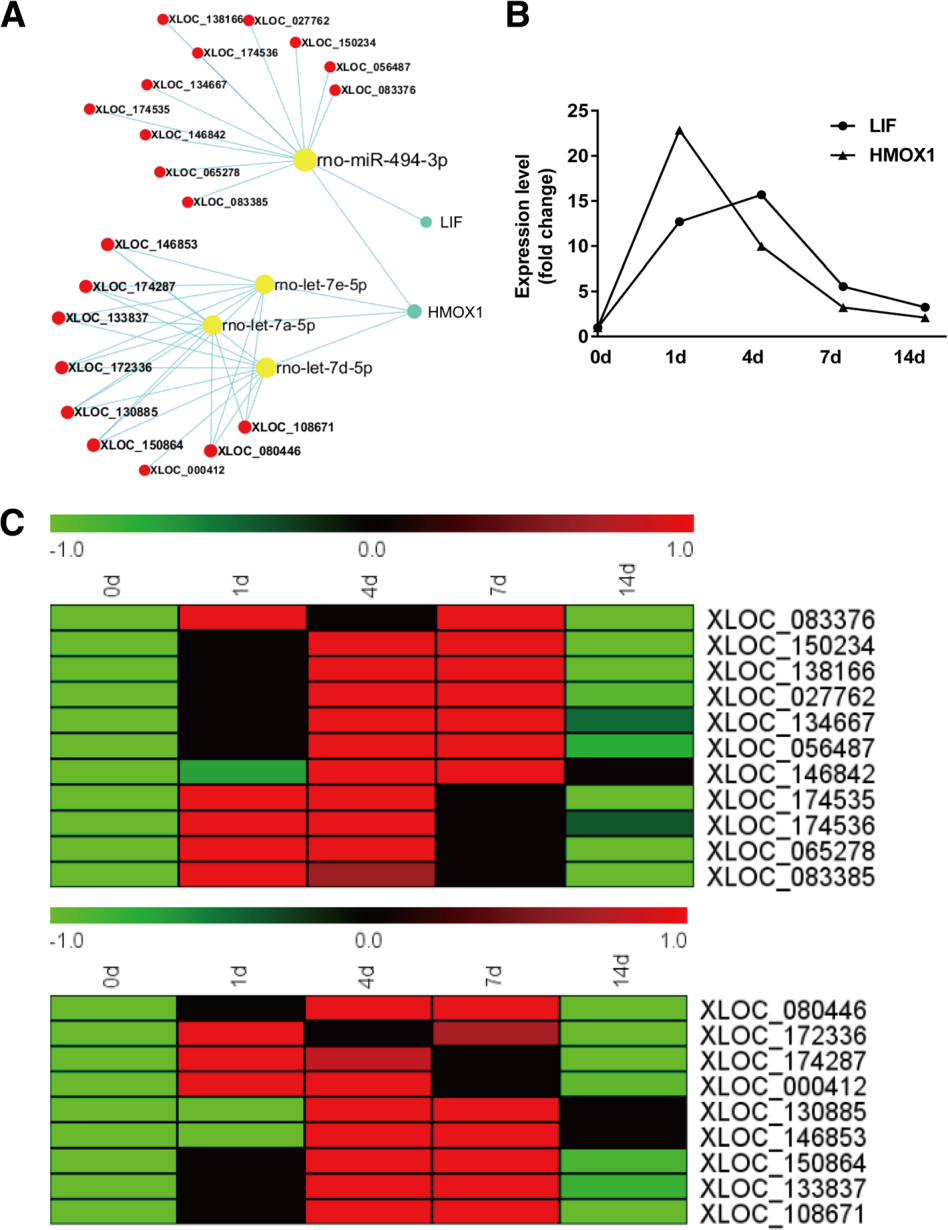
ceRNA network of LIF and HMOX1. **a** Interactions of RNAs in LIF and HMOX1-associated ceRNA network. **b** Expression levels of mRNAs LIF and HMOX1 from RNA sequencing outcome. **c** Heatmap and hierarchical clustering of lncRNAs in LIF and HMOX1-associated ceRNA network. Red color indicated up-regulation while green color indicated down-regulation

Enriched GO term “negative regulation of cell proliferation” contained three validated genes leukemia inhibitory factor (LIF), heme oxygenase (decycling) 1 (HMOX1), and peripheral myelin protein 22 (PMP22). Therefore validated genes in this GO term were further investigated. Outcomes from miRWalk database suggested that in GO term “negative regulation of cell proliferation”, LIF interacted with miR-494-3p, HMOX1 interacted with miR-494-3p, let-7e-5p, let-7a-5p, and let-7d-5p, and PMP22 interacted with miR-9a-5p and miR-29a-3p. Since both LIF and HMOX1 interacted with miR-494-3p, a network containing LIF, HMOX1, and their validated binding miRNAs was built (Fig. 5a). Moreover, we predicted lncRNAs that bound to miRNAs in the network by TargetScan, jointly analyzed mRNA-miRNA-lncRNA interactions, and constructed a LIF and HMOX1-associated ceRNA network (Fig. 5a). RNAs in the ceRNA network were further investigated. Sequencing outcomes suggested that both LIF and HMOX1 were up-regulated after nerve injury (Fig. 5b). The abundances of lncRNAs were also determined and demonstrated in heatmaps (Fig. 5c).

**Fig. 6:**
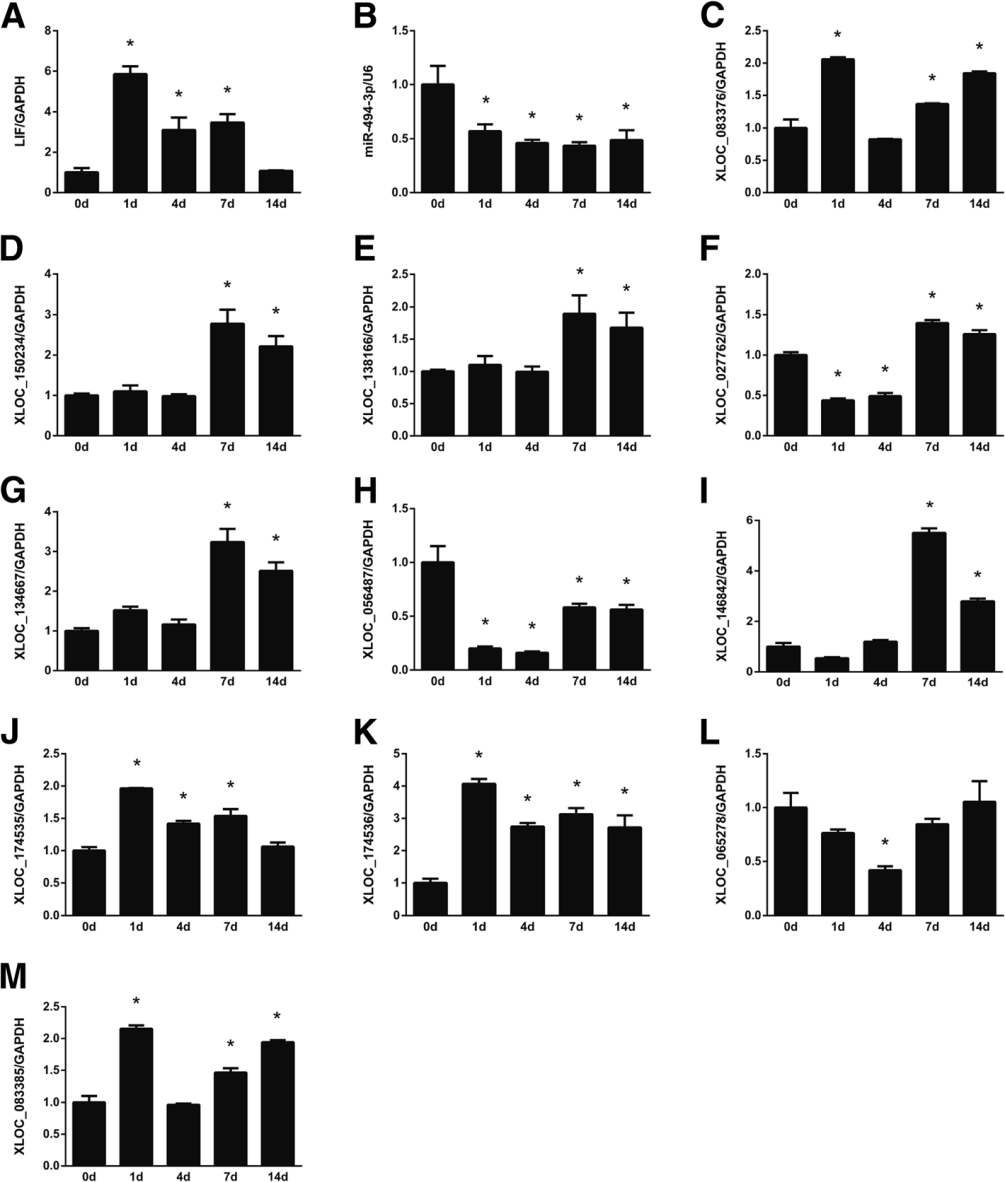
PCR validation of RNAs in the LIF-associated ceRNA network. The expression levels of (**a**) LIF, (**b**) miR-494-3p, (**c**) XLOC_083376, (**d**) XLOC_150234, (**e**) XLOC_138166, (**f**) XLOC_027762, (**g**) XLOC_134667, (**h**) XLOC_056487, (**i**) XLOC_146842, (**j**) XLOC_174535, (**k**) XLOC_174536, (**l**) XLOC_065278, and (**m**) XLOC_083385 at 0, 1, 4, 7, and 14 days after sciatic nerve injury were determined and normalized to reference RNA GAPDH. Numerical data were summarized from three independent experiments. *p-value< 0.05

The temporal expression levels of RNAs in the ceRNA network were further determined by quantitative RT-PCR. RT-PCR results showed that consistent with sequencing outcomes, the expression levels of LIF were increased after nerve injury (Fig. 6a). In contrast, the expression levels of miR-494-3p were decreased after nerve injury (Fig. 6b). The negative correlation of the expression patterns of LIF and miR-494-3p further demonstrated that LIF might be the mRNA target of miR-494-3p in the sciatic nerve segments after peripheral nerve injury. The expression levels of lncRNAs XLOC_083376, XLOC_150234, XLOC_138166, XLOC_027762, XLOC_134667, XLOC_056487, XLOC_146842, XLOC_174535, XLOC_174536, XLOC_065278, and XLOC_083385 were also determined. PCR results showed that XLOC_083376, XLOC_150234, XLOC_138166, XLOC_134667, XLOC_146842, XLOC_174535, XLOC_174536, XLOC_065278, and XLOC_083385 were mainly up-regulated after nerve injury, XLOC_056487, and XLOC_065278 were down-regulated, while XLOC_027762 was first down-regulated and then up-regulated (Fig. 6c–m).

**Fig. 7:**
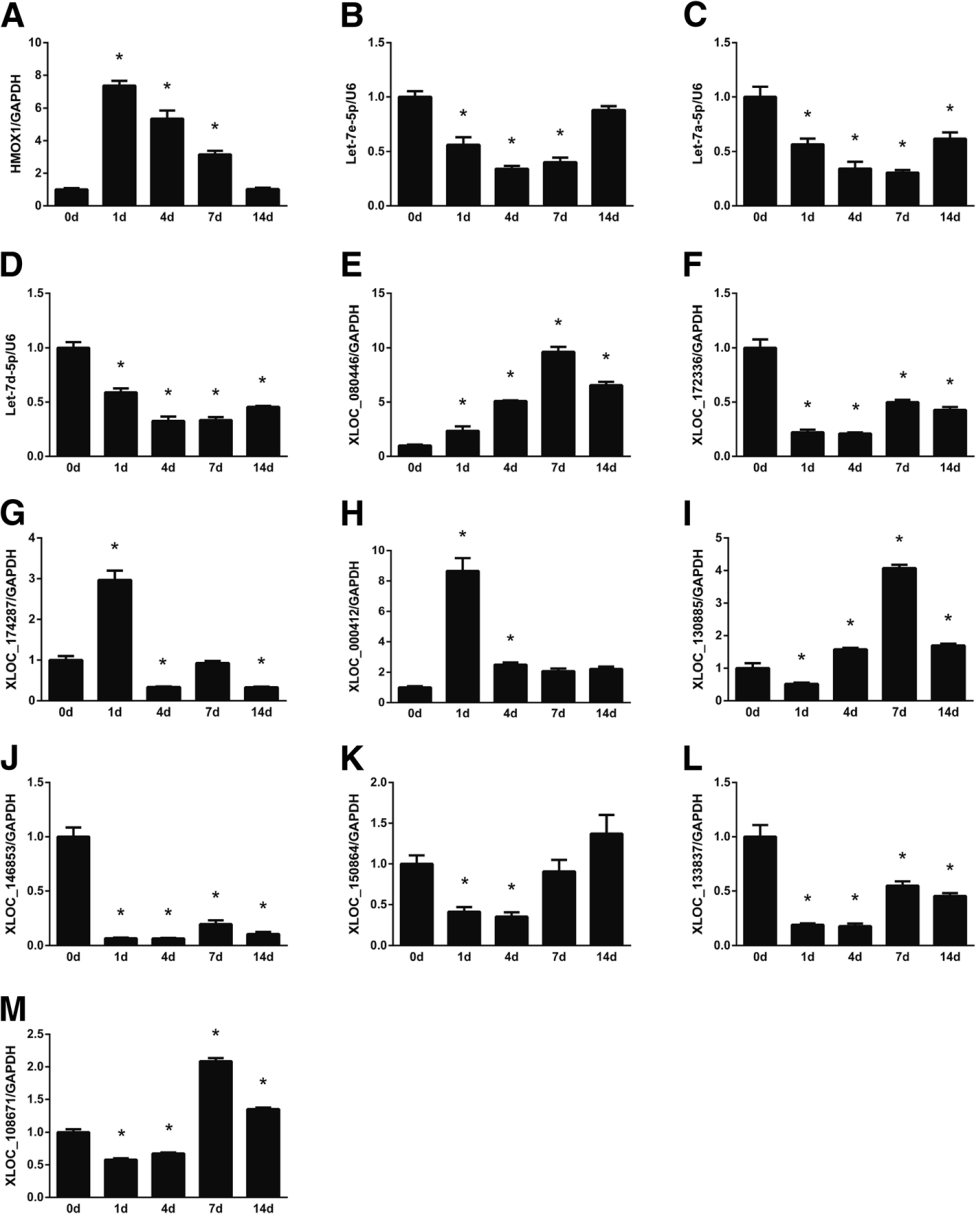
PCR validation of RNAs in the HMOX1-associated ceRNA network. The expression levels of (**a**) HMOX1, (**b**) let-7e-5p, (**c**) let-7a-5p, (**d**) let-7d-5p, (**e**) XLOC_080446, (f) XLOC_172336, (**g**) XLOC_174287, (**h**) XLOC_000412, (**i**) XLOC_130885, (**j**) XLOC_146853, (**k**) XLOC_150864, (**l**) XLOC_133837, and (**m**) XLOC_108671 at 0, 1, 4, 7, and 14 days after sciatic nerve injury were determined and normalized to reference RNA GAPDH. Numerical data were summarized from three independent experiments. *p-value< 0.05

Similarly, the abundances of RNAs in the HMOX1-let-7 ceRNA network were also determined. Following sciatic nerve injury, the expression levels of HMOX1 and let-7 (let-7e, let-7a, and let-7d) were increased and decreased, respectively (Fig. 7a–d). Together with observation in Fig. 6b, RT-PCR outcomes demonstrated that temporal expression changes of HMOX1 were inversely associated with those of miR-494-3p, let-7e, let-7a, and let-7d. Determination of lncRNA expression patterns showed that XLOC_080446, XLOC_174287, XLOC_000412, XLOC_130885, and XLOC_150864 were up-regulated, XLOC_172336, XLOC_146853, and XLOC_133837 were down-regulated, while XLOC_150864 and XLOC_108671 were first down-regulated and then up-regulated expressed.

## Discussion

In the current study, we used RNA deep sequencing and bioinformatic analysis to investigate molecular changes following peripheral nerve injury. Previously, by comparing the expressions of RNAs at different time points to their expressions at 0 day and by setting a threshold of fold change> 2 or < − 2 and FDR < 0.001, we identified 13,721, 14,321, 14,745, and 6979 differentially expressed RNAs at 1, 4, 7, and 14 days after nerve crush [14]. To screen out RNAs with extreme changes, here, we increased the threshold to fold change> 10 or < − 10 and FDR < 0.001 and discovered 957, 886, 590, and 444 significantly differentially expressed RNAs at 1, 4, 7, and 14 days after nerve crush, respectively. Moreover, we separately counted the numbers of differentially expressed mRNAs and lncRNAs and found that about 1/3 of differentially expressed RNAs were lncRNAs. The large amount of differentially expressed lncRNAs may further affect tons of mRNAs since lncRNAs could modulate the expressions of many mRNAs in multiple levels, including transcriptional regulation, post-transcriptional regulation, and epigenetic modification [17–19].

Euclidean distance, hierarchical clustering, and principal component analysis outcomes demonstrated that RNA expressions in the uninjured 0 day group were distinct from RNA expressions in the lesioned sciatic nerves. A comparison of RNA expressions at different time points after sciatic nerve injury showed that the expression profiles of RNAs in 1 day were also obviously different from those at later time points. GO annotation showed that a large number of GO cellular component, molecular function, and biological process terms were significantly enriched at 1 day after nerve crush while relatively smaller numbers of GO terms were enriched at later time points. Some inflammatory and immune response-related GO biological process terms (e.g., neutrophil chemotaxis, inflammatory response, and immune response) were kept activated at all time points. This was consistent with our previous observations of the importance of inflammatory and immune response after nerve injury [14, 20]. Enriched signaling pathways were also examined by KEGG pathway analysis. Our previous microarray analysis of the distal sciatic nerve segments showed that cytokine-cytokine receptor interaction and neuroactive ligand-receptor interaction were critical signaling pathways during Wallerian degeneration [21–25]. Here, we found that in the entire lesioned nerve segments, these two signaling pathways were also significantly involved. Some other signaling pathways, for example, chemokine signaling pathway, were demonstrated to be activated in the entire lesioned nerve segments but not the distal nerve segments. It implied that these signaling pathways might be important for nerve regrowth from the proximal nerve segments.

Besides these bioinformatic analyses, in the current study, we also jointly analyzed differentially expressed mRNAs and lncRNAs, identified the correlation of differentially expressed mRNAs and lncRNAs, clustered co-expressed mRNAs and lncRNAs, and performed functional analysis of co-expressed mRNAs and lncRNAs. Enriched GO terms and KEGG pathways in each cluster were identified and functional terms with validated mRNAs were further selected for the construction of ceRNA networks. There existed eight enriched GO terms (negative regulation of cell proliferation, cytosol, positive regulation of angiogenesis, response to hypoxia, response to nicotine, extracellular space, plasma membrane, and protein homodimerization activity) with more than one validated mRNAs in cluster 1 and one enriched GO term (neuron projection) with more than one validated mRNAs in cluster 2. We then selected GO term “negative regulation of cell proliferation”, identified bound miRNAs of validated genes in the GO terms by using miRWalk database, predicted interacted lncRNAs by using TargetScan software, and constructed LIF and HMOX1-associated ceRNA network.

Emerging studies have shown that miRNAs are key regulators in many physiological and pathological processes [26–28]. It has been demonstrated that many miRNAs were differentially expressed after peripheral nerve injury [29, 30]. Dysregulated miRNAs regulate Schwann cell proliferation, migration, and myelination and affect peripheral nerve regeneration [31–34]. Notably, the biological effects of miRNAs can be modulated by mRNAs, transcribed pseduogenes, lncRNAs, and circRNAs through the competitive binding to the miRNA response elements [35–38].

**Fig. 8:**
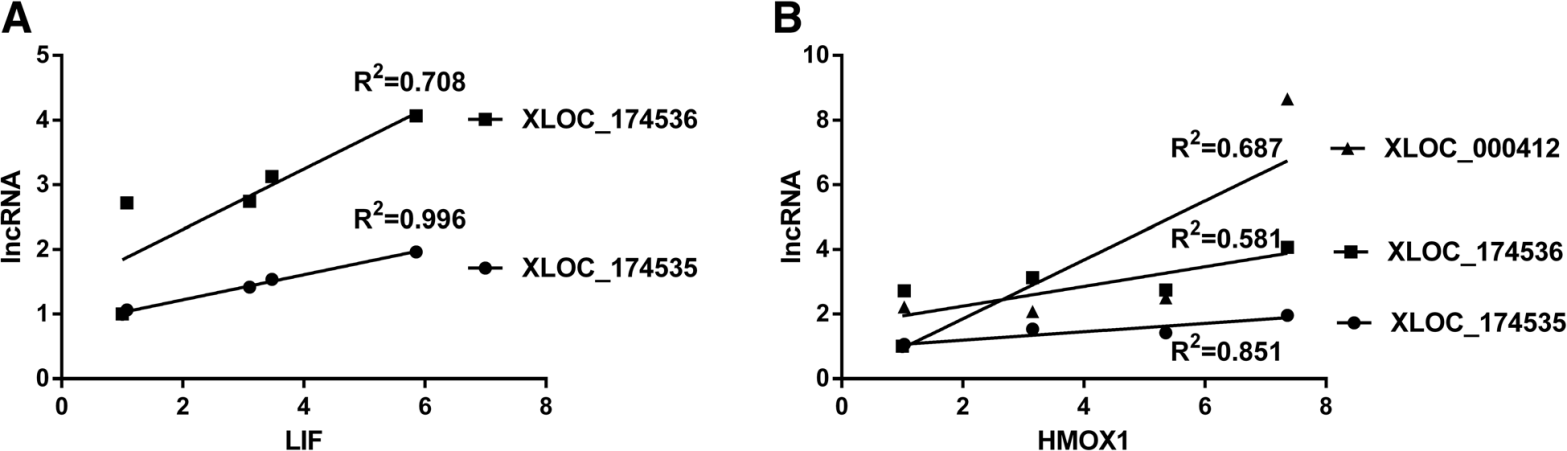
Correlation of mRNA and lncRNA expressions. **a** Expression correlation of LIF and lncRNAs XLOC_174535 and XLOC_174536. **b** Expression correlation of HMOX1 and lncRNAs XLOC_174535, XLOC_174536, and XLOC_000412. R^2^ was calculated by linear regression

As far as we know, till now, there is no study about lncRNA-associated ceRNAs in peripheral nerve repair and regeneration. Here, we used computational methods to discover correlations between mRNAs and lncRNAs and used miRWalk and TargetScan algorithm to explore mRNA-miRNA-lncRNA interactions. The temporal expression levels of mRNAs and lncRNAs in the constructed LIF and HMOX1-associated ceRNA network were also determined and demonstrated in line charts and heatmaps. Furthermore, the expression levels of mRNAs, miRNAs, and lncRNAs in the ceRNA network were validated by RT-PCR. Sequencing and RT-PCR results showed that both the expressions of LIF and HMOX1 were up-regulated after nerve injury. In contrast, the expressions of miR-494-3p, let-7e-5p, let-7a-5p, and let-7d-5p, validated bound miRNAs of LIF and HMOX1, were down-regulated. This, from the aspect of RNA expression, further supported that miR-494-3p, let-7e-5p, let-7a-5p, and let-7d-5p might regulate LIF and HMOX1 after peripheral nerve injury. Moreover, RT-PCR results showed that lncRNAs XLOC_174535, XLOC_174536, and XLOC_000412 were up-regulated after nerve injury, this was consistent with the expressions of LIF and HMOX1 and opposite with the expressions of miR-494-3p, let-7e-5p, let-7a-5p, and let-7d-5p. The correlations between the expressions of mRNAs and these lncRNAs were further calculated by linear regression (Fig. 8). The R^2^ of LIF expression and XLOC_174535 expression was 0.996 and the R^2^ of LIF expression and XLOC_174536 expression was 0.708 (Fig. 8a). The high R^2^ value suggested that LIF is positively correlated with lncRNAs XLOC_174535 and XLOC_174536, indicating that in LIF ceRNA network, lncRNAs XLOC_174535 and/or XLOC_174536 might sponge miR-494-3p and regulate LIF expression. Similarly, linear regression calculation showed that the R^2^ of HMOX1 expression and XLOC_174535 expression was 0.851 while the R^2^ of HMOX1 expression and XLOC_174535 expression was a little bit lower (0.581) (Fig. 8b). Therefore, in the HMOX1-miR-494-3p-lncRNA ceRNA network, lncRNA XLOC_174535 might sponge miR-494-3p and regulate HMOX1 expression. In HMOX1-let-7e-5p/let-7a-5p/let-7d-5p-lncRNA ceRNA network, the R^2^ of HMOX1 and XLOC_000412 was 0.687 (Fig. 8b), telling that lncRNA XLOC_000412 might sponge let-7e-5p/let-7a-5p/let-7d-5p and regulate HMOX1 expression. Luciferase assay could be performed to further examine the relationships of RNAs in the ceRNA network.

On the other hand, it is worth noting that our validation outcomes showed that the expression profiles of some lncRNAs were conflicting with sequencing outcomes. Many factors, including adequate replication, the quality of RNA, different efficiencies of reverse transcriptases, varied priming methods, and data normalization differences, can affect quantitative results and may lead to inconsistent results between outcomes from high-throughput analysis and RT-PCR validation [39]. This inconsistency was also observed in other studies [40–42]. Morey et al. calculated the direction of change in expression (up-regulation or down-regulation) by microarray and PCR and showed that the direction of change in expression was in agreement for 72.9% of samples [39]. Here, we found that in all tested 20 lncRNAs, 5 lncRNAs (XLOC_056487, XLOC_065278, XLOC_172336, XLOC_146853, and XLOC_133837) showed conflicting directions (down-regulated instead of up-regulated after nerve injury). The ratio of the direction of change in expression (25% disagreement) in our current study was similar as Morey’s calculation. In addition, we examined the sequencing RPKM reads of tested mRNAs and lncRNAs (Additional file 6: Table S6) and found that compared with the PRKM reads of HMOX1 and LIF, the PRKM reads of many lncRNAs were much lower. Genes with low absolute expression levels normally had larger changes of having inconsistent validation results [39]. Therefore, it is possible that the accuracy of lncRNAs with lower RPKM reads may be influenced by their lower expression levels.

In summary, in the current study, we studied the temporal changes of mRNAs and lncRNAs in the lesioned sciatic nerve segments at 0, 1, 4, 7, and 14 days after nerve crush and detected enriched GO terms and KEGG pathways. Moreover, for the first time, we elucidated the correlations of differentially expressed mRNAs and lncRNAs and delineated functional landscapes of mRNA-miRNA-lncRNA ceRNA network following peripheral nerve injury. Our current study expanded our knowledge about the molecular basis of peripheral nerve injury, provided insights of the potential regulations of non-coding RNAs, and offered promising prospects of the clinical treatment of peripheral nerve injury.

## Additional files

Additional file 1: **Table S1.** List of primer pairs for RT-PCR. (XLSX 19 kb)

Additional file 2: **Table S2.** List of significantly differentially expressed RNAs in the sciatic nerve segments after injury. RNAs with fold change> 10 or < − 10 and FDR < 0.001 as compared with 0 day control were considered as significantly differentially expressed. Gene ID, gene symbol, log_2_Ratio, fold change, *p*-value, and FDR of significantly differentially expressed mRNAs and lncRNAs were listed. (XLSX 379 kb)

Additional file 3: **Table S3.** List of enriched GO terms of significantly differentially expressed mRNAs in the sciatic nerve segments after injury. (XLSX 124 kb)

Additional file 4: **Table S4.** List of enriched GO terms and KEGG pathways of co-expressed mRNAs and lncRNAs. (XLSX 86 kb)

Additional file 5: **Table S5.** List of enriched GO terms of co-expressed mRNAs and lncRNAs with validated mRNAs. (XLSX 23 kb)

Additional file 6: **Table S6.** RPKM reads of mRNAs and lncRNAs in the sciatic nerve segments after injury. Expression levels of mRNAs (HMOX1 and LIF) and lncRNAs (XLOC_138166, XLOC_150234, XLOC_083376, XLOC_174287, XLOC_146853, XLOC_065278, XLOC_130885, XLOC_000412, XLOC_083385, XLOC_027762, XLOC_146842, XLOC_174535, XLOC_108671, XLOC_174536, XLOC_172336, XLOC_056487, XLOC_134667, XLOC_133837, XLOC_150864, and XLOC_080446) in the sciatic nerve segments at 0, 1, 4, 7, and 14 days after injury were demonstrated as RPKM reads. (XLSX 12 kb)

## Abbreviations

ANOVA: One-way analysis of variance
ceRNA: Competing endogenous RNA
circRNA: Circular RNA
DAVID: Database for Annotation, Visualization, and Integrated Discovery
FDR: False discover rate
GO: Gene ontology
HMOX1: Heme oxygenase (decycling) 1
KEGG: Kyoto Enrichment of Genes and Genomes
LIF: Leukemia inhibitory factor
lncRNA: Long non-coding RNA
miRNA: MicroRNA
PMP22: Peripheral myelin protein 22
RPKM: Reads per kilobase transcriptome per million mapped reads
SD: Sprague-Dawley
